# Use of Electronic Patient Record Systems for Rapid Response to an MHRA Public Assessment Report: Retrospective Observational Study

**DOI:** 10.2196/81355

**Published:** 2026-07-03

**Authors:** Dylan Whitaker, Matthew Wilson, Steve Harris, Yogini H Jani

**Affiliations:** 1 Department of Critical Care, University College London Hospitals NHS Foundation Trust, 250 Euston Rd, London, United Kingdom; 2 Department of Anaesthesia and Perioperative Medicine, University College London Hospitals NHS Foundation Trust, London, United Kingdom; 3 Division of Surgery and Interventional Science, University College London, London, United Kingdom; 4 Institute of Health Informatics, University College London, London, United Kingdom; 5 Centre for Medicines Optimisation Research and Education, School of Pharmacy, University College London and University College London Hospitals NHS Foundation Trust, 250 Euston Rd, London, United Kingdom, 44 20 3456 7890; 6 School of Pharmacy, University College London, London, England, United Kingdom

**Keywords:** electronic health records, electronic data processing, learning health system, analgesics, opioid, patient safety, medical informatics

## Abstract

**Background:**

Digital health data and infrastructure facilitate rapid analysis to provide actionable data, thereby fulfilling the principles of a learning health system. In response to a report from the UK Medicines and Healthcare Products Regulatory Agency (MHRA), a rapid service evaluation was carried out to identify patterns of modified-release (MR) opioid use after elective surgery.

**Objective:**

We aimed to describe the prescribing patterns of MR opioids, methods to repurpose existing infrastructure, and the experience of collaboration between clinical and research teams using shared data pipelines.

**Methods:**

A retrospective case-control study was conducted at a tertiary care organization across multiple hospital sites in London, United Kingdom. Prescription and administration data for adult patients undergoing elective surgery between March 31, 2019, and June 20, 2025, were extracted from a standardized research data pipeline within 4 weeks of the publication of the MHRA report. Patients were screened for MR opioid prescriptions in the postoperative period and at hospital discharge. Counts and proportions of encounters in which MR opioids were administered or prescribed were evaluated across the study period. Reflections on the application of the infrastructure for this purpose were also documented.

**Results:**

Of 126,882 elective surgeries screened, 102,879 (81.1%) met the eligibility criteria. Over the study period, patients received a new MR opioid prescription after 7525 (7.3%) of the 102,879 eligible encounters, with 2438 (2.4%) encounters receiving a new MR opioid prescription at hospital discharge. Postoperative administration of MR opioids and prescribing at discharge have declined since 2020. As a result of this study, a new context-aware alert system was developed to monitor and reduce MR opioid prescribing in this surgical cohort. Reflections on the implementation experience demonstrated how collaboration between clinical and research teams in conjunction with integrated and seamless research pipelines allowed rapid knowledge generation. Key issues raised were the difficulty of validation between parallel data extraction systems and how the two different teams compared nonequitable data points and results.

**Conclusions:**

Mature digital and analytical infrastructure within health care institutions can enable swift evaluation of local practices in the context of national medication safety alerts. This can shorten action response times and improve patient care but requires close collaboration between clinicians and research teams. Shared infrastructure between teams across the learning health system improves data quality and provides easy access to the key users. Further work is needed to understand the benefits and challenges of infrastructure built for other use cases and the effectiveness of the intervention.

## Introduction

On March 12, 2025, the UK Medicines & Healthcare Products Regulatory Agency (MHRA) published a public report recommending the removal of licenses for modified-release (MR) opioids (morphine and oxycodone in the UK context) for the treatment of acute postoperative pain [1]. The report addressed concerns from expert consensus groups (the Royal College of Anaesthetists, Faculty of Pain Medicine, Centre for Perioperative Care, Safe Anaesthesia Liaison Group, and the Medicines Safety Improvement Programme) regarding the safe use of MR opioids for the management of acute postsurgical pain [2].

Each year, the NHS performs approximately 1.5 million elective major surgeries, of which 48% of patients experience moderate to severe pain within the first 24 hours [3,4]. Initiation of opioid medications following surgery is an important pathway to persistent opioid use; 0.2% of surgical patients develop opioid misuse within 1 year, and this risk is tripled in patients discharged from hospital with an opioid prescription [5,6]. There is growing evidence of harm from the use of MR opioids, particularly regarding the risk of opioid-induced ventilatory impairment and long-term dependence [7-9]. Additionally, the MHRA report builds on both the 2020 international multidisciplinary consensus recommendation, “Long-active opioids should not be used routinely for acute postoperative pain,” [10] and the 2024 Association of Anaesthetists and British Pain Society consensus statement [11].

In 2024, the MHRA issued 27 medication alerts and reports ranging from updated information on side effects to contextual changes in response to evolving evidence and guidelines, as well as the removal of licenses for specific medication indications [1]. Following publication, responsibility for enacting change in response to these alerts is passed to individual hospitals and primary care providers, who must ensure that practice changes are implemented to maintain safe, high-quality patient care [12-14].

Digitally mature institutions can leverage existing infrastructure to allow rapid evaluation of and response to medication safety alerts, shortening the lag between MHRA notifications and implementation of changes in patient care. This rapid cycle of improvement fulfills the principles of a learning health system (LHS), addressing priority action area 2 (digital maturity) of the Health Foundation and Health Data Research UK report [15].

In this study, we aimed to do the following:

- Describe patterns of MR opioid prescribing following elective surgery
- Repurpose existing technical, ethical, and governance infrastructure to rapidly evaluate current MR opioid prescribing against the proposed licensing change and plan a digital intervention for reducing inappropriate MR opioid prescribing
- Describe the experience and challenges of collaboration between clinical and research teams using separate data sources and data extraction pipelines

## Methods

### Existing Infrastructure

University College London Hospitals NHS Foundation Trust (UCLH) has used the Epic electronic patient record (Epic Systems) since 2019. During this time, parallel data science infrastructure has been developed to facilitate the use of routinely collected patient data for secondary research, both extracted directly from the Epic data warehouses and from a separate research-ready platform in the Observational Medical Outcomes Partnership (OMOP) common data model (CDM) format [16,17]. These data sources are supported by the Experimental Medicines Application Platform, which facilitates access within existing UCLH IT security frameworks [18]. Version-controlled software for this infrastructure above is publicly available where privacy and intellectual property considerations permit [19,20]. Project-specific analysis code is publicly available on GitHub [21].

### Study Design

We analyzed data for all patients undergoing elective surgery between March 31, 2019, and June 20, 2025, identifying new MR opioid prescriptions in the postoperative period and at hospital discharge. Patients aged <18 years on the date of admission, those with no interoperative or inpatient medications, those undergoing procedures under local, topical, or regional anesthesia, and those who had not been discharged by the study end date were excluded. New prescriptions were identified based on the absence of a pre-existing medication prescription in the admission drug history. Analysis was performed using R software (version 4.4.1; R Foundation for Statistical Computing), with counts and proportions presented [22].

To reflect on the provision of data and validation of differing data sources, clinical teams and research teams met to discuss their requirements. Iterative cycles of development of the data pipeline were undertaken and reviewed in these meetings, and vignettes were captured for issues of clinical validation.

### Ethical Considerations

This project builds on research infrastructure designed and implemented for a research program on perioperative analgesic use. The wider research program was granted ethics approval via the UCLH Data Access Process for Research (Integrated Research Application System ID 299136). The data extraction and analysis for this specific study were performed under a service evaluation registration #136) to provide rapid feedback to the opioid stewardship team on historical and current use of MR opioid medications in this cohort. The data were deidentified. There was no requirement for informed consent, and no compensation was provided to participants.

## Results

### Data Analysis

**Figure 1.**
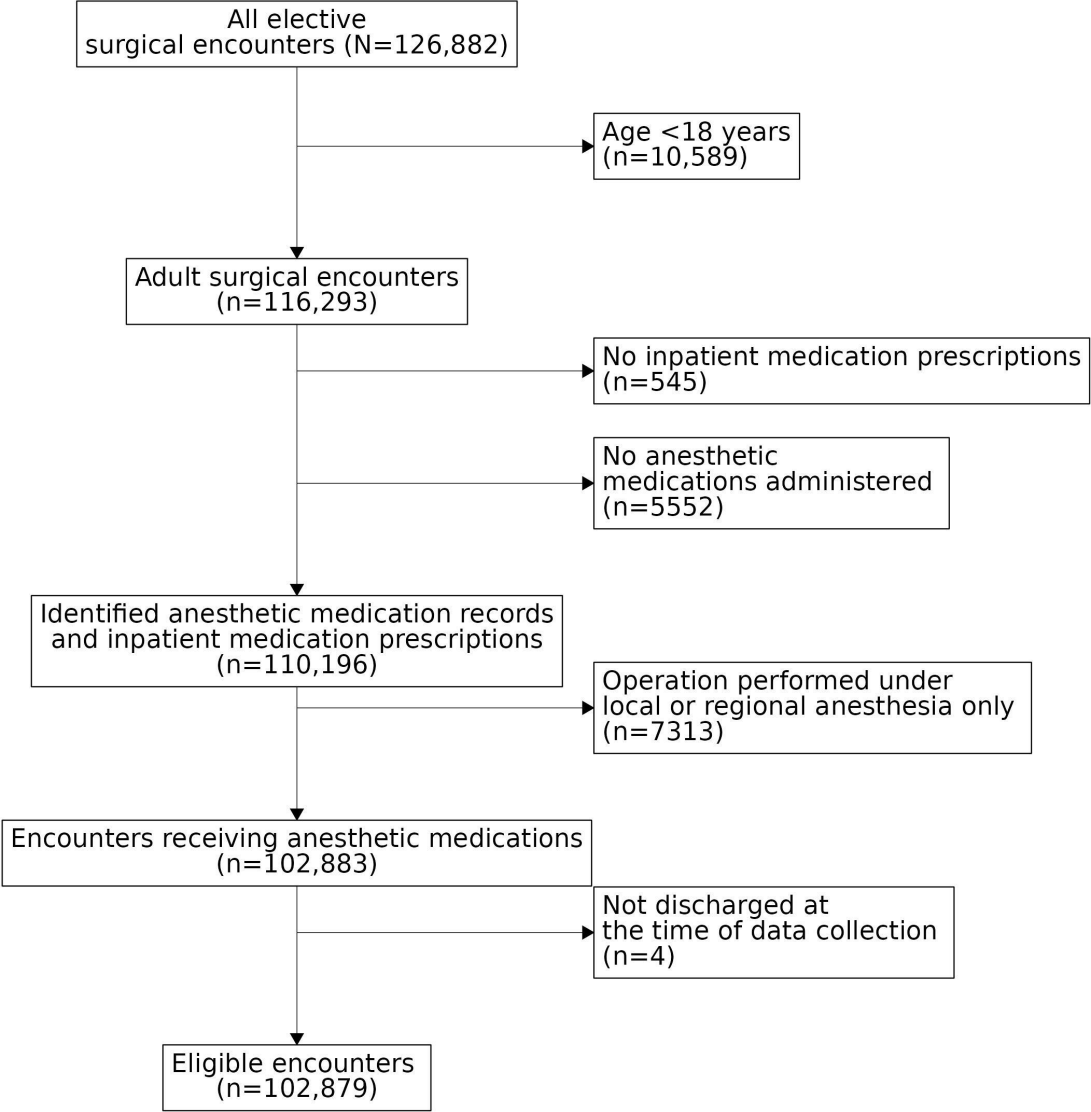
CONSORT (Consolidated Standards of Reporting Trials) diagram.

A total of 126,882 elective surgery encounters were identified during the study period, of which 102,879 (81.1%) met the eligibility criteria (Figure 1). Patient characteristics are described in Table 1.

**Table 1.** Patient and encounter characteristics (n=102,879).

Characteristics	Patients
Age (years), mean (SD)	52.17 (17.44)
Sex, n (%)	
Female	53,965 (52.5)
Male	48,900 (47.5)
Missing data	14 (<0.1)
Gender, n (%)	
Female	4668 (4.5)
Male	3569 (3.4)
Other	33 (<0.1)
Choose not to disclose	20 (<0.1)
Missing data	94,589 (91.9)
Ethnicity, n (%)	
Asian, Asian British, or Asian Welsh	7995 (7.8)
Black, Black British, Black Welsh, Caribbean, or African	7228 (7)
Missing data	23,182 (22.5)
Mixed or multiple ethnic groups	2377 (2.3)
Other ethnic group	6310 (6.1)
White	55,787 (54.2)
American Society of Anesthesiologists grade, n (%)	
1	18,659 (18.1)
2	58,713 (57.1)
3	23,987 (23.3)
4	712 (0.7)
5	5 (<0.1)
Missing data	803 (0.8)
Surgical specialty, n (%)	
Breast surgery	1995 (1.9)
Ear, nose, and throat	11,684 (11.4)
General surgery	11,285 (11)
Neurosurgery	9254 (9)
Obstetrics and gynecology	15,494 (15.1)
Oral and maxillofacial surgery	8334 (8.1)
Orthopedic surgery	11,846 (11.5)
Other	1685 (1.6)
Pediatric surgery	221 (0.3)
Thoracic surgery	3306 (3.2)
Urology	26,734 (26)
Vascular surgery	1041 (1)

Between 2019 and 2025, 7.3% (n=7525) of encounters were associated with a postoperative prescription for MR morphine or oxycodone, representing n=7081, 8.8% individual patients. In 6.9% (n=7144) of the total encounters where an MR opioid was prescribed, there was no preoperative MR opioid prescription associated with the patient. Overall, 2.4% (n=2438) of encounters received a new MR opioid prescription on discharge from hospital.

**Figure 2.**
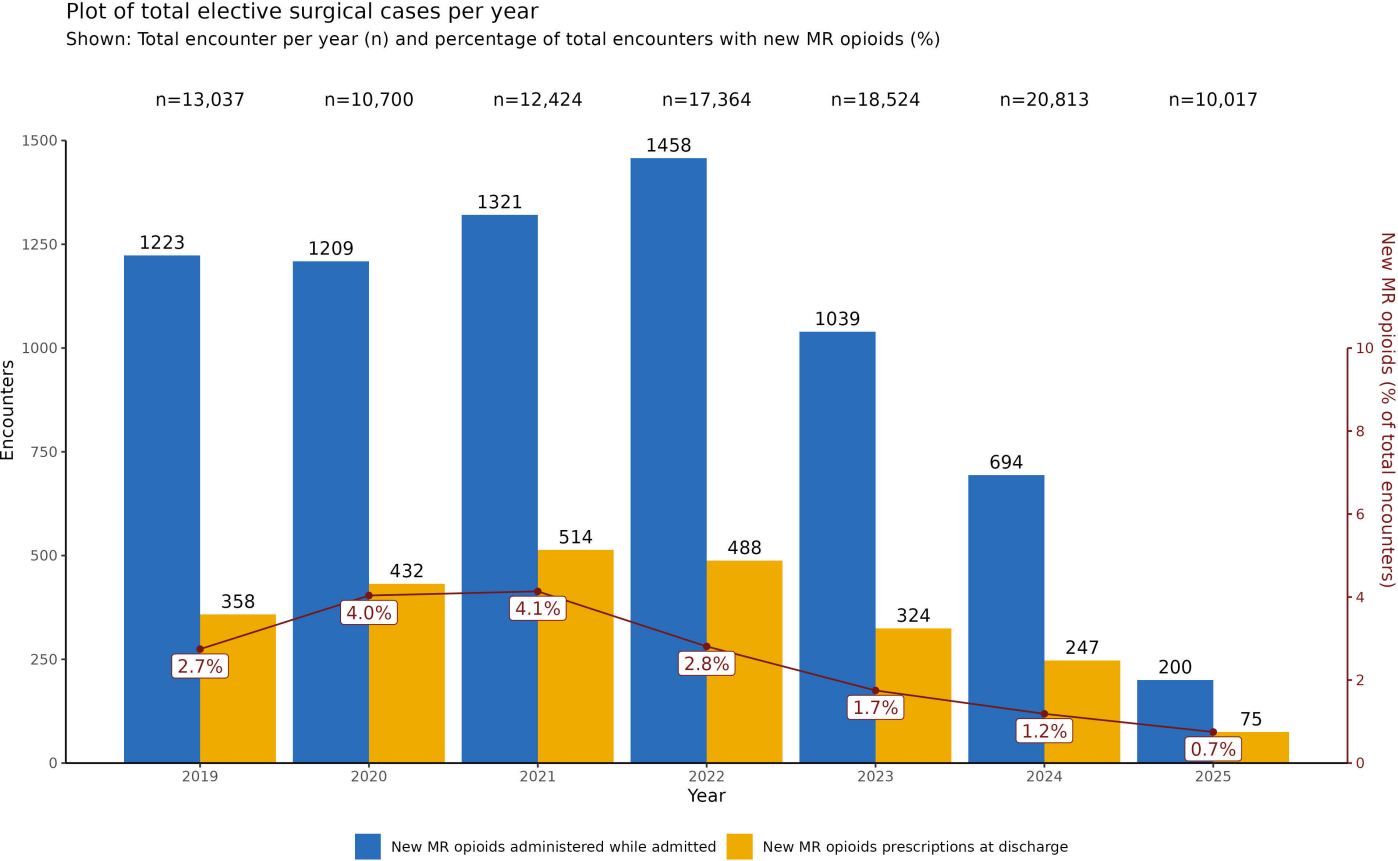
Bar chart showing the annual number of admissions where patients in which patients were administered a new modified-release (MR) opioid during admission (blue bars). Annual admissions in which patients were prescribed a new MR opioid at discharge are shown by the yellow bars. The red line indicates the percentage of total eligible admissions with new MR opioid prescription at discharge. The total number of eligible admissions for each year is shown above the bars.

When analyzed over time, overall prescribing rates had decreased prior to the MHRA notification; however, use of these medications continues (Figure 2).

### Experiences of the Implementation

The pivotal benefit of the integrated data infrastructure was the speed from MHRA notification to understanding and response. Study design, submission of the data access request, and sourcing the data were easy and rapidly performed, allowing multiple stages to progress in parallel. In this case, results were prepared within 4 weeks of the initial publication, allowing early engagement with the opioid stewardship team to co-design and implement digital and educational interventions to reduce MR opioid prescribing.

The availability of automated extraction of data in a direct Epic format and OMOP CDM format allowed for a broad range of data. This facilitated collaboration between clinicians and research teams through a shared understanding of the data and opportunities therein. Prior to this study, the clinical team highlighted that they had been requesting data on MR opioid use for more than 9 months to no avail, whereas during this period, this feature has been built out in the research dataset for wider perioperative research.

Cross-validation of variables proved a challenge, exposing several “analogous” variables between different data views and formats that were calculated in differing ways or from differing sources and thus not equitable or equivalent. This required much work to trace data lineage and ensure that variables were indeed comparable between formats. One such example was the mislabeling of a specific fentanyl prescription, used for boluses in recovery, as the medication type “morphine sulphate.” Examination of the medication administration record showed that the drug-to-dose relationships appeared correctly; however, when calculating oral morphine equivalence values, a systematic error was introduced by the automated matching of the fentanyl dose to the conversion value of 3 for morphine (as opposed to 0.2 for fentanyl) [23]. The impact of this error was difficult to trace given the wide range of possible routes to extract and calculate metrics, such as oral morphine equivalence, across different analysis pipelines. This highlighted the need to ensure harmony and equivalence between the values viewed by clinicians built-in electronic health record (EHR) tools, the values extracted by business intelligence teams from EHR data warehouses, and the values generated by researchers using data extracts (such as the OMOP CDM).

Another significant issue was raised about the recording of total intravenous anesthesia. The charting of regular intraoperative medications and total intravenous anesthesia medications differed within the EHR interface, leading to different approaches to extract the data across different pipelines and, consequently, different summed values for the total dose of remifentanil from the same anesthetic charts, depending on the data pipeline used.

## Discussion

### Summary

In this report, we demonstrate how existing digital and electronic patient record infrastructure can be used to rapidly respond to medication safety reports and changes in medication licensing.

Developing a shared infrastructure to support both secondary research using patient data and rapid audit and quality improvement evaluations is a foundational component of modern LHS, for which there are good examples in the literature [15,24,25].

Our work used an existing research data infrastructure for examining perioperative analgesic strategies to rapidly evaluate patterns of MR opioid prescribing and highlight problem areas for targeted intervention. Establishing a dedicated set of tools to extract, transform, clean, and store data, known as a data pipeline, and analysis workflow for perioperative analgesia, was highly efficient as it could be used to evaluate the success of future interventions to reduce prescribing and facilitate “active monitoring,” allowing long-term surveillance of prescribing practices.

Despite the range of data extraction pipelines available to research and information teams, generating hypotheses and evidence still requires extensive discussions with clinical teams. Even with steps designed to smooth this process, several clinical teams remain dependent on either built-in EHR reporting tools (which offer limited data granularity and customization) or custom data requests from clinical data analysts (a resource-intensive approach with limited generalizability). The 9-month wait for data requested on MR opioids by the clinical team, while related work was already being undertaken within the research team, highlighted gaps in data access, data quality, and the “siloed” working practices common across large health care organizations [26]. Furthermore, cross-team communication is essential to ensure that lessons on data quality and validation are cascaded across different pipelines to avoid diverging datasets and ensure comparability.

From March 2025 to March 2026, there were 4 other MHRA safety public assessment reports with recommendations for prescribers of valproate, fluoroquinolone antibiotics, isotretinoin and gabapentinoids, benzodiazepines, and z-drugs [27-30]. While this report focused specifically on MR opioids in the perioperative context, the need for high-quality prescribing and administering data that are structured, validated, and accessible is vital for designing solutions to current and future patient safety alerts.

Implementing digital LHSs requires both technological and analytical maturity to facilitate rapid responses to changing evidence on treatment safety and effectiveness. Furthermore, it requires an organizational culture in which clinicians with subject matter expertise can work closely with informatics and implementation science experts to successfully improve care. To fully realize the principles of an LHS, we must ensure that data are easily accessible to the teams requiring it most.

### Limitations

The study cohort was derived from a single large teaching hospital in London. The population is reflective of the patient cohort in London and may have limited generalizability to MR opioid prescribing patterns in other contexts. Although the extraction of preadmission medications was accurate with respect to the EHR data, a number of publications have highlighted possible inaccuracies in the underlying quality of medication reconciliation processes [31,32].

### Conclusions

We have shown how routinely collected electronic patient data may be used to evaluate a national medication safety alert and identify priority areas for further interventions. Our data pipeline works alongside Epic and, therefore, is relatively provider-agnostic. Hospitals with comprehensive electronic patient records should foster close connections between data science teams, business intelligence teams, and clinicians to curate and analyze their high-quality data to improve care.

Further work will focus on establishing the generalizability and broader applications for the infrastructure developed.

## Abbreviations

CDM: common data model
EHR: electronic health record
LHS: learning health system
MHRA: Medicines & Healthcare Products Regulatory Agency
MR: modified-release
OMOP: Observational Medical Outcomes Partnership
UCLH: University College London Hospitals NHS Foundation Trust

## References

[1] (2025). Modified release opioids and treatment of post-operative pain: public assessment report. https://assets.publishing.service.gov.uk/media/67d01193d5ec5ed9e09f5ef4/Modified_Release_Opioids_and_Treatment_of_Post-operative_Pain.pdf.

[2] Quinlan J, Levy N, Laycock H (2023). Letter to MHRA - opioid stewardship. Safe Anaesthesia Liaison Group.

[3] Moonesinghe SR, McGuckin D, Martin P (2022). The Perioperative Quality Improvement Programme (PQIP patient study): protocol for a UK multicentre, prospective cohort study to measure quality of care and outcomes after major surgery. Perioper Med (Lond).

[4] Walker EM, Bell M, Cook TM (2016). Patient reported outcome of adult perioperative anaesthesia in the United Kingdom: a cross-sectional observational study. Br J Anaesth.

[5] Brat GA, Agniel D, Beam A (2018). Postsurgical prescriptions for opioid naive patients and association with overdose and misuse: retrospective cohort study. BMJ.

[6] Clarke H, Soneji N, Ko DT, Yun L, Wijeysundera DN (2014). Rates and risk factors for prolonged opioid use after major surgery: population based cohort study. BMJ.

[7] Quinlan J, Levy N, Lobo DN, Macintyre PE (2022). No place for routine use of modified-release opioids in postoperative pain management. Br J Anaesth.

[8] Liu S, Athar A, Quach D (2023). Risks and benefits of oral modified-release compared with oral immediate-release opioid use after surgery: a systematic review and meta-analysis. Anaesthesia.

[9] Liu S, Patanwala AE, Naylor JM (2023). Impact of modified-release opioid use on clinical outcomes following total hip and knee arthroplasty: a propensity score-matched cohort study. Anaesthesia.

[10] Levy N, Quinlan J, El-Boghdadly K (2021). An international multidisciplinary consensus statement on the prevention of opioid-related harm in adult surgical patients. Anaesthesia.

[11] El-Boghdadly K, Levy NA, Fawcett WJ (2024). Peri-operative pain management in adults: a multidisciplinary consensus statement from the Association of Anaesthetists and the British Pain Society. Anaesthesia.

[12] Levy N, Lobo DN, Macintyre PE (2025). An obituary for postoperative use of modified-release opioids. Anaesthesia.

[13] Snowdon A, Hussein A, Olubisi A, Wright A (2024). Digital maturity as a strategy for advancing patient experience in US hospitals. J Patient Exp.

[14] Curtis HJ, MacKenna B, OpenSAFELY Collaborative (2021). OpenSAFELY: impact of national guidance on switching anticoagulant therapy during COVID-19 pandemic. Open Heart.

[15] Hardie T, Horton T, Thornton-Lee N, Home J, Pereira P (2022). Developing learning health systems in the UK: priorities for action. https://www.health.org.uk/reports-and-analysis/reports/developing-learning-health-systems-in-the-uk-priorities-for-action.

[16] Harris S, Bonnici T, Keen T, Lilaonitkul W, White MJ, Swanepoel N (2022). Clinical deployment environments: five pillars of translational machine learning for health. Front Digit Health.

[17] University College London Hospitals NHS OMOP dataset. Health Data Research Gateway.

[18] Data Access Process for Research (DAP-R). UCLH Biomedical Research Centre.

[19] SAFEHR-data/omop_es. GitHub.

[20] SAFEHR-data/emap. GitHub.

[21] Whitaker D DW10/mhra_mr_opioids. GitHub.

[22] R Core Team (2024). R: a language and environment for statistical computing. R Foundation for Statistical Computing.

[23] Nielsen S, Degenhardt L, Hoban B, Gisev N (2016). A synthesis of oral morphine equivalents (OME) for opioid utilisation studies. Pharmacoepidemiol Drug Saf.

[24] Horwitz LI, Kuznetsova M, Jones SA (2019). Creating a learning health system through rapid-cycle, randomized testing. N Engl J Med.

[25] Foley T, Horwitz L, Zahran R (2021). Realising the potential of learning health systems. https://learninghealthcareproject.org/realising-the-potential-of-learning-health-systems/.

[26] Lau RS, Boesen ME, Richer L, Hill MD (2024). Siloed mentality, health system suboptimization and the healthcare symphony: a Canadian perspective. Health Res Policy Syst.

[27] (2025). Valproate: managing reproductive risks in male patients under 55. https://www.gov.uk/government/publications/valproate-managing-reproductive-risks-in-male-patients-under-55.

[28] (2026). Review of the impact of recommendations for the prescribing of isotretinoin. https://www.gov.uk/government/publications/review-of-the-impact-of-recommendations-for-the-prescribing-of-isotretinoin.

[29] (2026). Improving information supplied with gabapentinoids (pregabalin/gabapentin), benzodiazepines and z-drugs. https://www.gov.uk/government/publications/improving-information-supplied-with-gabapentinoids-pregabalingabapentin-benzodiazepines-and-z-drugs.

[30] (2025). Review of risk minimisation for disabling and potentially long-lasting/irreversible side effects associated with fluoroquinolone antibiotics. https://tinyurl.com/56nxfvrm.

[31] Shah C, Hough J, Jani Y (2020). Medicines reconciliation in primary care: a study evaluating the quality of medication-related information provided on discharge from secondary care. Eur J Hosp Pharm.

[32] Urban R, Armitage G, Morgan J, Marshall K, Blenkinsopp A, Scally A (2014). Custom and practice: a multi-center study of medicines reconciliation following admission in four acute hospitals in the UK. Res Social Adm Pharm.

